# Student-initiated near-peer information panel on residency application process

**DOI:** 10.36834/cmej.53161

**Published:** 2020-07-15

**Authors:** Nicholas Sequeira, Alon Coret, Brandon Tang, Flora Jung

**Affiliations:** 1 MD Program, Faculty of Medicine, University of Toronto, Ontario, Canada; 2 Department of Medicine, University of British Columbia, BC, Canada

## Implication Statement

We describe a student-run, near-peer information panel at the University of Toronto on the residency application process. Similar panels currently exist at multiple medical schools across Canada. Previous papers indicate that students find peer-to-peer and near-peer advising to be particularly useful for CaRMS, electives, and clerkship-related topics. This event aimed to provide pre-clerkship students with information on strategies for CaRMS applications, elective selection, excelling during clerkship, and maintaining wellness. Topics were selected based on interests expressed in a pre-event student survey; therefore, this panel could be easily modified to suit the specific needs of students at other schools.

## Déclaration des répercussions

Nous décrivons un panel d’information de quasi-pairs dirigé par des étudiants à l’Université de Toronto sur le processus de demande de résidence. Des panels similaires existent actuellement dans de nombreuses facultés de médecine au Canada. Des articles antérieurs indiquent que les étudiants trouvent que les conseils entre des pairs ou des quasi-pairs sont particulièrement utiles pour les thèmes relatifs au CaRMS, aux stages à option et à des sujets reliés à l’externat. Cet évènement visait à fournir aux étudiants des renseignements sur les stratégies en lien avec les demandes au CaRMS, le choix des stages à option, la performance durant l’externat et le maintien du bien-être. Les thèmes ont été sélectionnés selon les intérêts exprimés dans un sondage pré-évènement auprès des étudiants ; par conséquent, ce panel pourrait être facilement modifié pour convenir aux besoins particuliers des étudiants d’autres facultés.

Completing a residency program is a necessary step on the path to becoming a practicing clinician, taking a minimum of two years and as long as seven years. Regretfully, the process by which students ‘match’ to a residency – better known as CaRMS (Canadian Resident Matching Service) – is a common source of anxiety.^1^ While most applications are successful, a growing percentage of medical graduates do not match to a residency program, necessitating a year-long wait until the next application cycle.^2^ Given these implications, students across the country are becoming increasingly eager to understand the CaRMS process earlier in training. A prior study showed that advice from peers was highly influential in helping students prepare CaRMS applications and make electives decisions.^3^ Another study indicated that 77% of medical students felt near-peer information sessions on succeeding in clerkship were helpful.^4^ As such, medical students at the University of Toronto implemented an annual ‘CaRMS panel’ to provide pre-clerkship students with relevant and well-informed advice on this issue. Other medical schools, including Saskatchewan, Western, McMaster, Queen’s, McGill, and Memorial, hold similar events.

The CaRMS panel was organized annually in April from 2016-2018. It was held on campus in the evening. The study was exempt from ethical review by the University of Toronto Human Research Ethics Program. The panel consisted of 10 graduating students who recently matched to various residency programs. Panelists were selected based on matched specialty to represent a wide variety of common specialties. The panel always included at least one representative from family medicine, internal medicine, surgery, psychiatry, pediatrics, and OB/GYN. Most often, panelists who matched to emergency medicine, anesthesiology, and radiology were also included. In 2018, this event was expanded to include 2 ‘unmatched’ panelists. Topics were informed by themes that emerged from a pre-event student survey (n = 70) and were iteratively adjusted based on particular interests each year. They included strategies for CaRMS applications, elective selection, how to be a ‘good clerk’, and maintaining wellness. After a 50-minute discussion and a 10-minute Question & Answer (Q&A) period, students had the opportunity to speak with panelists individually.

Following the 2016 event, students (n = 35) completed an optional online survey rating the event’s impact on their preparedness for and understanding of CaRMS, electives, and clerkship on a 5-point Likert scale (Table 1). Eighty-four percent (84%) of students agreed that information provided was insufficiently covered in formal settings. Qualitative feedback indicated that students desired even greater diversity in the panel, including representatives from smaller specialties.

**Table 1:** Post-event student opinions on the impact of the CaRMS panel on their preparedness for and understanding of electives, CaRMS, and clerkship

Statement	Disagree	Neutral	Agree	Strongly Agree
I feel more prepared for electives	3%	27%	60%	10%
I feel more prepared for CaRMS	3%	42%	42%	13%
I feel more prepared for clerkship	3%	43%	43%	10%
I have a better understanding of the electives process	0%	7%	71%	23%
I have a better understanding of the CaRMS process	0%	13%	67%	20%
I have a better understanding of how to succeed in clerkship	0%	19%	68%	13%
Information from this panel is not covered in the medical school curriculum	0%	16%	65%	19%

Survey results indicate that a majority of students felt the panel discussion and Q&A were valuable and informative. As the survey was distributed immediately after the event, it is possible that the panel had long-term implications that were not reflected in the results. Next steps include a follow-up survey prior to CaRMS examining the relationship between CaRMS panel attendance and preparedness for the CaRMS process, as well as further integration of the event with UGME-directed career counseling initiatives. Furthermore, in future iterations of the event, we will explicitly identify and include panelists who matched to their second-choice specialty to further expand panel diversity.
